# Virtual decoupling to break the simplification versus resolution trade-off in nuclear magnetic resonance of complex metabolic mixtures

**DOI:** 10.5194/mr-2-619-2021

**Published:** 2021-08-10

**Authors:** Cyril Charlier, Neil Cox, Sophie Martine Prud'homme, Alain Geffard, Jean-Marc Nuzillard, Burkhard Luy, Guy Lippens

**Affiliations:** 1 Toulouse Biotechnology Institute (TBI), Université de Toulouse, CNRS, INRAE, INSA, Toulouse, France; 2 Université de Reims Champagne-Ardenne (URCA), UMR-I 02 SEBIO (Stress Environnementaux et Biosurveillance des milieux aquatiques), Moulin de la Housse, Reims, France; 3 Université de Reims Champagne Ardenne, CNRS, ICMR UMR 7312, 51097 Reims, France; 4 Institute for Biological Interfaces 4 – Magnetic Resonance, Karlsruhe Institute of Technology (KIT), Herrmann-von-Helmholtz-Platz 1, 76344 Eggenstein-Leopoldshafen, Germany; a present address: Université de Lorraine, CNRS, LIEC, 57000, Metz, France

## Abstract

The heteronuclear single quantum correlation (HSQC) experiment developed by Bodenhausen and Ruben (1980) in the early days of modern nuclear magnetic resonance (NMR) is without a doubt one of the most widely used experiments, with applications in almost every aspect of NMR including metabolomics. Acquiring this experiment, however, always implies a trade-off: simplification versus resolution. Here, we present a method that artificially lifts this barrier and demonstrate its application towards metabolite identification in a complex mixture. Based on the measurement of clean in-phase and clean anti-phase (CLIP/CLAP) HSQC spectra (Enthart et al., 2008), we construct a virtually decoupled HSQC (vd-HSQC) spectrum that maintains the highest possible resolution in the proton dimension. Combining this vd-HSQC spectrum with a J -resolved spectrum (Pell and Keeler, 2007) provides useful information for the one-dimensional proton spectrum assignment and for the identification of metabolites in *Dreissena polymorpha* (Prud'homme et al., 2020).

## 1 Introduction

The recognition that a given nucleus was characterized by a specific chemical shift value depending on its exact environment in a molecule (Proctor and Yu, 1950; Dickinson, 1950) ushered nuclear magnetic resonance (NMR) from its initial discovery in a nuclear physics department (Purcell et al., 1946; Bloch et al., 1946) into the chemistry sphere. Magnetic shielding of nuclei was identified at the origin of the phenomenon (Ramsey, 1950) and representative chemical shift values of the different protons in organic molecules were rapidly established (Arnold et al., 1951; Bernstein and Schneider, 1956). The further recognition of homonuclear scalar coupling (the “ J -coupling”) patterns (Gutowsky and McCall, 1951) increased the information content, and notions as singlet, doublet, triplet, etc. were rapidly used to characterize the individual proton lines in the NMR spectra and to assist the identification of the molecule under study. Realizing that these same J couplings could be used to transfer magnetization from one spin to another, in 1971, Jeener proposed an indirect acquisition scheme to reconstruct a 2-D map (Jeener and Alewaeters, 2016), whereby the off-diagonal peaks connect different protons through their J coupling. The homonuclear correlation spectroscopy (COSY) experiment was born (Aue et al., 1976), and many two- and higher-dimensional homonuclear pulse sequences would adopt a similar principle.

One problem with proton ( ${}^{1}$ H) NMR is the limited range of chemical shift values (typically 10 ppm) with line widths on the order of magnitude of 1 Hz, leading to a crowded spectrum especially when complex mixtures are studied. The carbon ( ${}^{13}$ C) chemical shift range is significantly larger ( ∼ 200 ppm), making it an attractive alternative to characterize a (mixture of) molecule(s). However, its low natural abundance ( ∼ 1.1 %), inherently poorer sensitivity with its 4-fold lower gyromagnetic ratio and long relaxation times increase the required acquisition times by a factor of 10 ${}^{6}$ or higher when a similar S/N as for the proton spectrum is desired. The introduction of a two-dimensional (2-D) heteronuclear single quantum correlation (HSQC) spectrum by Bodenhausen and Ruben in 1980 has literally been a game changer in the NMR field (Bodenhausen and Ruben, 1980). In this pioneering publication, cited 3000 times as of the time of writing, “the detection of NMR spectra of less sensitive nuclei coupled to protons was shown to be significantly improved by a two-dimensional Fourier transform technique involving a double transfer of polarization”. Building on the ideas of 2-D NMR (Jeener and Alewaeters, 2016; Aue et al., 1976) and magnetization transfer between a heteronucleus and proton by their scalar coupling (Maudsley and Ernst, 1977; Morris and Freeman, 1979), polarization transfer from the proton to the insensitive heteronucleus in the excitation step and back to proton for the detection led to a tremendous gain in sensitivity. The HSQC experiment was born, and although initially described for a proton directly linked to its amide nitrogen, it was readily applied to other nuclei such as ${}^{199}$ Hg (Roberts et al., 1980), ${}^{113}$ Cd (Live et al., 1985), ${}^{31}$ P (Bolton and Bodenhausen, 1982) and ${}^{13}$ C (Bendall et al., 1983). The HSQC experiment applied to the latter ${}^{1}$ H– ${}^{13}$ C pair has not only solved sensitivity problems associated with the direct ${}^{13}$ C 1-D spectrum but also greatly increased the information content of both individual ${}^{1}$ H and ${}^{13}$ C spectra as it connects a carbon signal directly to its proton binding partner. The success story of the HSQC spectrum was born and formed the basis for a myriad of 2- to n -D spectra used from protein NMR to the analysis of metabolomic samples.

Indeed, beyond synthetic chemistry, NMR has found its place in the realm of metabolomics (Emwas et al., 2019; Wishart, 2019), and together with mass spectrometry, it is the method of choice for characterizing complex mixtures such as biofluids. If throughput is required, the 1-D ${}^{1}$ H spectrum remains the general workhorse, whose assignment can be based on database searches, on the spiking of relevant standards within the sample or on analysis of the corresponding 2-D spectra acquired on one or more samples. The ${}^{1}$ H– ${}^{13}$ C HSQC spectrum proposed by Bodenhausen and Ruben is again a crucial element in the latter approach, as it spreads out the proton signals according to the ${}^{13}$ C chemical shift of its attached carbons and allows connection of both in the same time. The use of 2-D experiments in the field of metabolomics, despite inherent longer experimental times, has grown extensively, in part due to the introduction of ultra-fast acquisition (Tal and Frydman, 2010), ASAP (acceleration by sharing adjacent polarization) techniques (Schätzlein et al., 2018) and non-uniformly sampling (NUS) acquisition scheme (Guennec et al., 2014; Schlippenbach et al., 2018; Zhang et al., 2020). Based on these developments, the use of 2-D experiments for larger number of samples has become within reach and will probably place the ${}^{1}$ H– ${}^{13}$ C HSQC spectrum as a cornerstone of metabolomic studies by NMR. However, for quantitative studies, the 1-D proton spectrum with a long relaxation delay will remain the reference, as both limits on the relaxation time and variable J -coupling values make the HSQC spectrum inherently non-quantitative. Assignment of the 1-D spectrum on the basis of the HSQC spectrum and extracting quantitative information from the former is however one way to proceed.

In the initial HSQC paper, it was recognized that obtaining a single peak for the ${}^{15}$ N– ${}^{1}$ H pair requires decoupling both during the indirect detection (obtained by a single proton π pulse) and the direct proton detection (through a broadband decoupling scheme). However, for technical reasons related to the large spread of the ${}^{13}$ C resonances, decoupling during the direct acquisition period can lead to sample heating and/or probe arcing. One typically limits acquisition times to 100 ms, and especially with current high S/N cryogenically cooled probes, constructors limit the power delivery during this decoupling period (Bahadoor et al., 2021). As a result, the resolution in the ${}^{1}$ H dimension is limited and the homonuclear coupling pattern of the protons cannot be observed, despite the fact that they carry valuable information.

Here, we explore the capacity to virtually decouple a HSQC spectrum without physical decoupling during the acquisition time, thereby removing all physical limits on the acquisition time. We reconstruct the decoupled spectrum by recording two coupled HSQC spectra: one with the direct ${}^{1}$ H– ${}^{13}$ C doublet in phase and one with the doublet in anti-phase. Comparable sequences aimed at measuring the ${}^{1}$ J coupling constant have been published before, especially in the framework of determining the residual dipolar couplings for samples partially oriented in the liquid phase (Andersson et al., 1998). However, here we use both spectra only to distinguish the up- and downfield components of the doublet, thereby providing the necessary information for shifting one component with its complex homonuclear coupling pattern to the central position. As a result, we obtain a HSQC spectrum with a high resolution in the proton dimension. Traces from it can be immediately superimposed on the 1-D ${}^{1}$ H spectrum or on a high-resolution J -resolved ( J -res) spectrum, thereby helping the assignment of the latter. We first demonstrate the approach on the spectrum of an isolated oligosaccharide and show its use on a zebra mussel (*Dreissena polymorpha*) hydrophilic extract that we recently studied in the framework of an ecotoxicological study (Prud'homme et al., 2020).

## 2 Methods

All experiments were performed on an Avance III 800 MHz spectrometer (Bruker BioSpin GmbH, Karlsruhe, Germany), equipped with a 1.7 mm triple-resonance HCP or a 5 mm quadruple resonance QCI-P (H/P-C/N/D) cryoprobe and were recorded at 298 K. Data were processed with TopSpin 4.0.8 (Bruker BioSpin GmbH, Karlsruhe, Germany). All proton spectra were referenced to the methyl proton of 3-trimethylsilylpropionic acid-d4 (TSP-d ${}_{4}$ ). ${}^{13}$ C chemical shifts were determined by indirect referencing (Markley et al., 1998).

Pulse sequences were initially tested on a sample of 1 mg of dextran oligosaccharide dissolved in 40 µ L of D ${}_{2}$ O plus 2 µ L of TSP-d ${}_{4}$ on the 1.7 mm cryoprobe. Clean in-phase and clean anti-phase (CLIP/CLAP) experiments were acquired with 16 384 ( ${}^{1}$ H) × 256 ( ${}^{13}$ C) time points, a spectral width of 12.0172 ppm ( ${}^{1}$ H) × 60 ppm ( ${}^{13}$ C) and NS = 8, DS = 16, RG = 512, d1 = 1.5 s, experimental time = 1 h 23 min 2 s (CLIP) and 1 h 22 min 39 s (CLAP). Data were transformed to a matrix of 16 384 ( ${}^{1}$ H) × 1024 ( ${}^{13}$ C) frequency points. ${}^{1}$ H– ${}^{13}$ C HSQC with decoupling was recorded with 4096 ( ${}^{1}$ H) × 256 ( ${}^{13}$ C) time points, a spectral width of 12.0172 ppm ( ${}^{1}$ H) × 60 ppm ( ${}^{13}$ C) and NS = 8, DS = 32, RG = 512, d1 = 1 s, experimental time = 43 min 2 s. Data were transformed to a matrix of 4096 ( ${}^{1}$ H) × 1024 ( ${}^{13}$ C) frequency points.

Hydrophilic extracts of zebra mussel were prepared a described in (Prud'homme et al., 2020). Briefly, 50 mg of biomass were dried prior to resuspension in 50 µ L of 100 mM potassium phosphate buffer dissolved in D ${}_{2}$ O supplemented with 1 mM sodium azide and 0.5 mM TSP-d ${}_{4}$ . From this solution, 40 µ L were then transferred into the NMR sample. CLIP/CLAP experiments were acquired with 16 384 ( ${}^{1}$ H) × 256 ( ${}^{13}$ C) time points, a spectral width of 13.9486 ppm ( ${}^{1}$ H) × 100 ppm ( ${}^{13}$ C) and NS = 64, DS = 64, RG = 512, d1 = 1.5 s, experimental time = 10 h 28 min 51 s (CLIP) and 10 h 25 min 48 s (CLAP). Data were transformed to a matrix of 16 384 ( ${}^{1}$ H) × 1024 ( ${}^{13}$ C) frequency points. The 1-D ${}^{1}$ H, ${}^{1}$ H– ${}^{13}$ C HSQC with decoupling and the J -resolved experiments were recorded on the 5 mm cryoprobe on a sample of 200 µ L. A high-resolution 1-D ${}^{1}$ H spectrum was acquired with 32 768 time points, a spectral width of 12.0172 ppm and NS = 64, DS = 4, RG = 8, d1 = 5.0 s. The ${}^{1}$ H– ${}^{13}$ C HSQC was recorded with 2048 ( ${}^{1}$ H) × 256 ( ${}^{13}$ C) time points, a spectral width of 13.9486 ppm ( ${}^{1}$ H) × 100 ppm ( ${}^{13}$ C) and NS = 64, DS = 64, RG = 912, d1 = 1 s, experimental time = 8 h 2 min 26 s. Data were transformed to a matrix of 2048 ( ${}^{1}$ H) × 512 ( ${}^{13}$ C) frequency points. The J -resolved experiment was recorded with 16 384 time points, a spectral width of 12.0172 ppm and NS = 128, DS = 64, RG = 912, d1 = 1.32 s for an experimental time of 18 h 1 min 29 s.

## 3 Results and discussion

As pointed out in the original paper of Bodenhausen and Ruben, without physical decoupling of the direct ${}^{1}$ H– ${}^{13}$ C interaction, the theoretical pattern of a peak in the direct dimension ( $F_{2}$ ) of the HSQC spectrum is a doublet, due to the heteronuclear coupling constant ( ${}^{1}J_{\mathrm{CH}}$ for a ${}^{1}$ H / ${}^{13}$ C spectrum). Each component of the doublet can moreover display a more or less complex coupling pattern due to homo- ( $J_{\mathrm{HH}}$ ) or heteronuclear (for example, proton–phosphorus coupling, $J_{\mathrm{HP}}$ ) coupling interactions involving the observed proton. The active ${}^{1}J_{\mathrm{CH}}$ coupling can be removed by heteronuclear spin decoupling during signal acquisition and renders more easily interpretable 2-D maps with a single cross peak per ${}^{1}$ H / ${}^{13}$ C pair. Decoupling, however, has its drawbacks, especially when the bandwidth to be decoupled increases. Although numerous approaches have been developed to decrease the delivered power (Kobzar et al., 2004; Kupče, 2020) and hence reduce potential sample heating, limiting the acquisition time remains the first option, be it at the detriment of resolution and hence loss of intrinsic coupling pattern. Ideally, one would want both – a decoupled ${}^{1}$ H spectrum with a single peak per ${}^{1}$ H / ${}^{13}$ C pair with a high resolution leaving the intricate coupling pattern of the proton intact.

Omitting the decoupling during the acquisition is the obvious solution to the heating problem but leads to a doubling of the number of peaks in the spectrum and thereby potentially increases spectral overlap. Moreover, the peaks resonate at ±J/2 Hz from their true chemical shift, whereby J is the active coupling at the origin of the cross peak and hence cannot directly be superimposed on the 1-D proton spectrum dominated by the contribution of the ${}^{12}$ C-linked protons. For isolated peaks, however, this doublet structure can be easily recognized, and shifting both components back to their central position solves the problem.

This reconstruction of the decoupled spectrum meets problems, however, when peak overlap becomes important. One issue is whether a given peak corresponds to the down- or upfield component of the doublet, necessitating to look to the left or right for its corresponding component. For this, however, solutions have long existed, notably in the endeavour to measure (small) coupling constants. Combining absorption/dispersion non-decoupled spectra has been used to measure coupling constants down to few hertz for well-resolved signals (Kessler et al., 1985; Oschkinat and Freeman, 1984). A 2-D X-filtered total correlation spectroscopy (TOCSY) (Wollborn and Leibfritz, 1992) or its corresponding 1-D version (Nuzillard and Bernassau, 1994) was proposed to improve the accuracy of the coupling constant measurement. Other experiments such as α / β -HSQC/heteronuclear multiple quantum coherence (HMQC) (Andersson et al., 1998) or α / β -HSQC-TOCSY (Koźmiński, 1999) were developed based on the concept of spin-state selection (Meissner et al., 1997) to selectively observe in each subspectrum a single component of the coupling multiplet. However, as these methods are not applicable without directly linked protons to the carbon, workarounds have been proposed using heteronuclear single quantum multiple bond correlation (HSQMBC) (Williamson et al., 2000) or HSQC spectra (Titman et al., 1989) supplemented with Carr–Purcell–Meiboom–Gill (CPMG) train pulses (Boros and Kövér, 2011; Kövér et al., 2006) to improve the intrinsic twisted linewidth due to the evolution of homonuclear proton–proton couplings. New pulse sequences were later developed to facilitate measurement of one-bond coupling constants by eliminating sources of line-shape distortion, such as the clean in-phase (CLIP)-HSQC experiment (Enthart et al., 2008). Acquiring the anti-phase magnetization in a separate clean anti-phase (CLAP)-HSQC experiment followed by addition/subtraction of both spectra yields high-resolution α and β -state subspectra. BEBOP (broadband excitation by optimized pulses)/BIBOP (broadband inversion by optimized pulses) (Kobzar et al., 2004; Luy et al., 2005) pulses on ${}^{13}$ C can be used to obtain uniform performance of the experiments across the large carbon spectral width. Long-range proton–carbon coupling constants have been measured with the CLIP-HSQMBC (Saurí et al., 2013), PIP-HSQMBC (Castañar et al., 2014) and CSSF-CLIP-HSQMBC (Moreno et al., 2019).

Proton multiplet patterns are often seen as a source of increased peak overlap that needs to be addressed by a myriad of pure-shift methods (Zangger and Sterk, 1997; Foroozandeh et al., 2014; Castañar and Parella, 2015). These have been combined with HSQC sequences to alleviate extraction of coupling constants (Timári et al., 2016). However, we believe that the information contained in these patterns can be crucial towards the assignment and identification of metabolites in complex mixtures. Herein, we combine the original CLIP/CLAP pulse sequences (Enthart et al., 2008) with virtual decoupling of a ${}^{1}$ H– ${}^{13}$ C spectrum based on automated recognition of the α and β states of the multiplets and subsequent back-shifting of the high-resolution lines to their true chemical shift. We thereby obtain both a single peak per ${}^{1}$ H / ${}^{13}$ C moiety and the required high resolution for identification of its J -coupling pattern.

**Figure 1:**
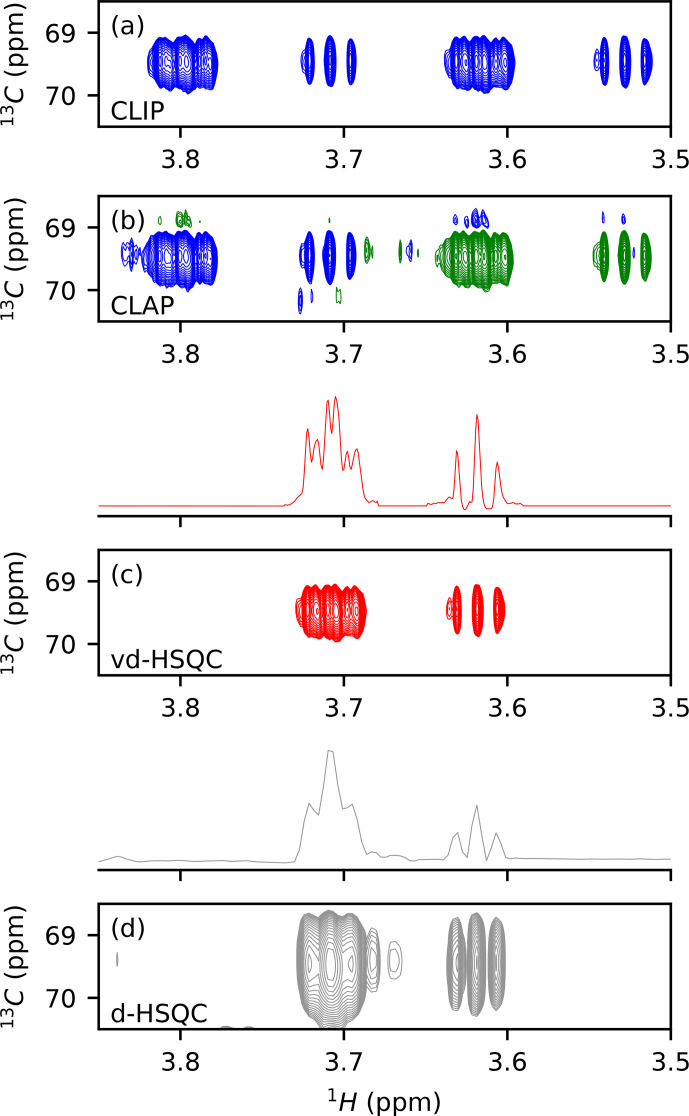
Application of virtual decoupling isolated dextran oligosaccharide. CLIP **(a)** and CLAP **(b)** are shown with positive contours in blue and negative contours in green. **(c)** Virtually decoupled HSQC. **(d)** Decoupled HSQC. The traces shown above panels **(c)** and **(d)** are extracted at the centre of the peak of the 2-D spectra.

We first tested the potential of virtual decoupling on an isolated dextran oligosaccharide. Both CLIP/CLAP experiments acquired under identical conditions led to two high-quality spectra, as shown in Figs. 1a, b and S1 in the Supplement. The CLIP experiment in which the anti-phase proton magnetization is refocused with a 180 ${}^{\circ }$ ${}^{13}$ C pulse during the final INEPT followed by a 90 ${}^{\circ }$ ${}^{13}$ C pulse prior to detection to remove any dispersive component shows two purely in-phase signals separated by the heteronuclear coupling constant ${}^{1}J_{\mathrm{CH}}$ . In the CLAP experiment, these two pulses are omitted, leading to the observation of the anti-phase doublet. The absence of decoupling during the acquisition in the CLIP/CLAP experiment allows the observation of each component of the doublets with high resolution (Fig. S1). We developed a semi-automatic Python procedure for the virtual decoupling that is based on the *nmrglue* package (Helmus and Jaroniec, 2013) and is usable within TopSpin 4. It can be downloaded from https://github.com/NMRTeamTBI/VirtualDecoupling (last access: 4 August 2021). While launching the script from TopSpin, the user will have several options that are detailed in the tutorial available online. First, taking advantage of the sign difference between the α and β components of the doublet in the CLAP experiment (Fig. S2), we reduce the resolution and hence the fine structure of both components by processing the CLAP spectrum with only 1024 points in the ${}^{1}$ H dimension, thereby intentionally destroying the high-resolution J -coupling pattern and obtaining just a single peak per component. Automatic peak detection is then performed twice: once on the positive components and once on the negative components of the spectrum and can be performed on the full spectral width or only for a user-defined region with a threshold that can be adapted if necessary. A clustering step based on ${}^{13}$ C chemical shifts obtains a pairwise selection of the signals. The central position of this pair is used to define the J/2 value for this particular ${}^{1}$ H / ${}^{13}$ C pair, thereby individualizing the back shift and avoiding the impossible use of a common value for all peaks. If exactly two signals of opposite sign are found with the same ${}^{13}$ C frequency (in digital points), the script will automatically bring the upfield component extracted from CLIP-HSQC spectrum with full resolution (processed with 16 384 points in the time domain) back to the centre of the doublet and thereby create a virtually decoupled HSQC (vd-HSQC) spectrum (Figs. 1c and S3). In the case of more than two signals identified at the same ${}^{13}$ C frequency value, a user interface will pop up and allow manual selection and clustering of the signals.

**Figure 2:**
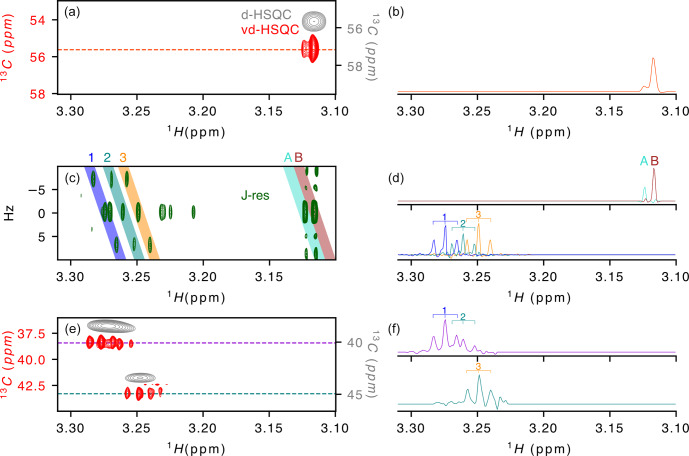
Application of virtual decoupling to a zebra mussel sample. **(a)** Overlay of the virtually decoupled HSQC (red) and decoupled HSQC (grey) and **(b)** 1-D ${}^{1}$ H trace extracted from the vd-HSQC at 55.623 ppm. **(c)** J -resolved spectrum with **(d)** the ${}^{1}$ H projections from the coloured areas on the 2-D spectrum and named A/B (top) and 1/2/3 (bottom). Coloured areas were extracted using the *zerter* command in TopSpin (Cox et al., 2021). **(e)** Virtually decoupled HSQC (red) and decoupled HSQC (grey) and **(f)** 1-D ${}^{1}$ H trace extracted from the vd-HSQC at 38.426 ppm (top) and 43.298 ppm (bottom). 1/2/3 correspond to the three doublets of doublets identified in areas 1/2/3 of the J -res experiment.

After developing the procedure on this simple oligosaccharide sample, we turned to a zebra mussel (*Dreissena polymorpha*) extract as representative of a more complex sample. We recently annotated the spectrum in the framework of a NMR-based metabolomic ecotoxicological study and identified and assigned a large number of metabolites that can be used as reporters of environmental health (Prud'homme et al., 2020). While the initial metabolomic study of the zebra mussel (Watanabe et al., 2015) was based on 1-D ${}^{1}$ H and 2-D ${}^{1}$ H– ${}^{13}$ C HSQC spectrum, our more recent work (Prud'homme et al., 2020) also included 2-D ${}^{1}$ H– ${}^{1}$ H, 2-D ${}^{1}$ H– ${}^{31}$ P and J -resolved spectra and led to the assignment of ∼ 50 % of the signals observed on the 1-D ${}^{1}$ H spectrum. To evaluate the value of virtual decoupling on such a complex sample, we measured the CLIP/CLAP spectra of the *D. polymorpha* whole-body polar extract in order to reconstruct the vd-HSQC and combined it with a high-resolution J -res spectrum. Here, we focus on the region of the trimethylamines from 3.1 to 3.3 ppm (Fig. 2). The J -resolved experiment shows the presence of two singlets around 3.12 ppm (peaks A and B in Fig. 2c). Because they almost overlap and moreover differ by a factor of 10 in intensity, the decoupled HSQC (Fig. 2a grey spectrum) only shows a single peak in which the two signals cannot be resolved. However, in the corresponding region of the virtually decoupled HSQC (Fig. 2a), the full proton pattern containing two signals is observed, confirming that both protons are linked to a carbon with identical chemical shift. The trace extracted at 55.623 ppm through the vd-HSQC (Fig. 2b) perfectly matches the trace extracted at 0 Hz in the J -res spectrum (Fig. 2d), in agreement with a negligible isotope shift of the ${}^{13}$ C nucleus on the proton chemical shift (Tiainen et al., 2010). This first example illustrates the ability of the vd-HSQC to separate two signals that cannot be resolved with a conventional decoupled HSQC (d-HSQC).

A second example of the vd-HSQC's capacity to assist and enhance the assignment is given by the three doublets of doublets (dd) highlighted by the numbers on the J -res spectrum (Fig. 2c). Using the *zerter* programme recently developed in our group (Cox et al., 2021) first to isolate and zero the singlet at 3.27 ppm and then extract the individual pseudo-triplets, each extracted trace shows the expected 1 : 2 : 1 pattern (lines 1, 2 and 3 in Fig. 2d). These ${}^{1}$ H 1-D traces can be used to search through the vd-HSQC spectrum to identify the corresponding ${}^{13}$ C resonance frequencies. Because of the high resolution of the vd-HSQC, the scan is not only based on the chemical shift value but equally on the proton–proton coupling pattern, thereby significantly enhancing the information content. Two patterns were identified at ${}^{13}$ C resonance frequencies of 38.426 and 43.298 ppm (Fig. 2e). In both cases, the vd-HSQC shows the correct ${}^{1}$ H frequency and the correct coupling pattern. Indeed, the trace extracted from the signal at the higher ${}^{13}$ C frequency (Fig. 2f) shows the presence of a 1 : 2 : 1 coupling pattern which fits that of the projected spectrum of the J -res. The peak identified at the lower ${}^{13}$ C frequency in the vd-HSQC shows two 1 : 2 : 1 patterns for which the ${}^{1}$ H trace can be matched with the projection of the J -res spectrum. This combined information enhances our confidence that the three dd signals, although closely together in proton chemical shift and coupling pattern, actually represent organic moieties that differ by 5 ppm in carbon chemical shift. If we compare this with a scan through the d-HSQC, assigning the broad peak at 38.426 ppm to the two dd signals seems more hazardous, especially as the singlet at 3.271 ppm could also be erroneously assigned to this proton frequency. The coupling pattern hence increases the information content and, when combined with a high-resolution J -resolved spectrum, can lead to accurate information on individual proton resonances.

**Figure 3:**
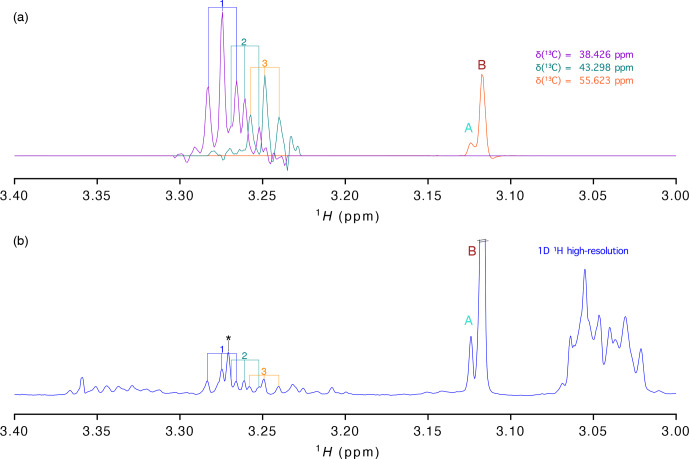
**(a)** ${}^{1}$ H traces extracted from the virtually decoupling at different ${}^{13}$ C chemical shifts and **(b)** 1-D ${}^{1}$ H spectrum with high resolution (acquired with 16 384​​​​​​​ points). Signals are labelled as in Fig. 2.

As stated before, presently most studies aiming at the analysis of a large number of samples are based on the high-resolution 1-D proton spectrum. To evaluate how well the information of our vd-HSQC spectrum can be transferred to the 1-D spectrum, we show in Fig. 3 the traces extracted from the vd-HSQC spectrum on top of the high-resolution 1-D spectrum of the *D. polymorpha* extract (Fig. 3). The two singlets at the ${}^{13}$ C frequency of 55.623 ppm can immediately be identified in the 1-D spectrum, and their corresponding intensities represent the concentrations of both entities if the 1-D spectrum is acquired with a sufficiently long relaxation delay. But even in the crowded region around 3.25 ppm, we can use the traces of the vd-HSQC to assign the individual pseudo-triplets and associate them with their corresponding ${}^{13}$ C values. Interestingly, the 1-D spectrum also contains the singlet at 3.271 ppm (indicated by an asterisk (*) in Fig. 3), but the combined information from the high-resolution vd-HSQC and J -res spectra immediately singles it out as such.

## 4 Conclusion

Beyond the time constraints associated with the 2-D nature of the spectrum, the use of the ${}^{1}$ H– ${}^{13}$ C HSQC in metabolomics is always associated with an NMR dilemma: broadband decoupling during acquisition greatly simplifies the spectra, but due to limitations in the duty cycle it leads to the loss of all information about proton multiplicities; no decoupling during acquisition leads to the observation of the complete proton multiplet pattern but doubles the number of resonances. Virtual decoupling of the spectrum is of great interest for this issue because it provides a single resonance that maintains the highest achievable resolution. Here, we explore the benefits of this approach and demonstrate that the virtually decoupled spectrum can be directly compared with the high-resolution 1-D proton spectrum, thereby helping in the process of metabolite identification over many samples. The methodology presented here, while being robust, is rather simplistic and will benefit from the rise of deep learning and artificial intelligence applications to NMR spectra (Karunanithy et al., 2021) to provide a new entry point into metabolite databases.

## Supplement

10.5194/mr-2-619-2021-supplement The supplement related to this article is available online at: https://doi.org/10.5194/mr-2-619-2021-supplement.

## Data Availability

The code written in Python for virtual decoupling and compatible within TopSpin 4 can be found on GitHub: https://github.com/NMRTeamTBI/VirtualDecoupling (https://doi.org/10.5281/zenodo.5163919, Charlier et al., 2021). A readme file guides potential users through the data used in the paper.

## References

[bib1.bib1] Andersson P, Nordstrand K, Sunnerhagen M, Liepinsh E, Turovskis I, Otting G (1998). Heteronuclear correlation experiments for the determination of one-bond coupling constants. J Biomol NMR.

[bib1.bib2] Arnold JT, Dharmatti SS, Packard ME (1951). Chemical Effects on Nuclear Induction Signals from Organic Compounds. J Chem Phys.

[bib1.bib3] Aue WP, Bartholdi E, Ernst RR (1976). Two-dimensional spectroscopy. Application to nuclear magnetic resonance. J Chem Phys.

[bib1.bib4] Bahadoor A, Brinkmann A, Melanson JE (2021). ${}^{13}$
C-Satellite Decoupling Strategies for Improving Accuracy in Quantitative Nuclear Magnetic Resonance. Anal Chem.

[bib1.bib5] Bendall MR, Pegg DT, Doddrell DM (1983). Pulse sequences utilizing the correlated motion of coupled heteronuclei in the transverse plane of the doubly rotating frame. J Magn Reson.

[bib1.bib6] Bernstein HJ, Schneider WG (1956). Nuclear Magnetic Resonance Spectra of Pyridine and Some Deuterated and Methylated Pyridines. J Chem Phys.

[bib1.bib7] Bloch F, Hansen WW, Packard M (1946). Nuclear Induction. Phys Rev.

[bib1.bib8] Bodenhausen G, Ruben DJ (1980). Natural abundance nitrogen-15 NMR by enhanced heteronuclear spectroscopy. Chem Phys Lett.

[bib1.bib9] Bolton PH, Bodenhausen G (1982). Resolution enhancement in heteronuclear two-dimensional spectroscopy by realignment of coherence transfer echoes. J Magn Reson.

[bib1.bib10] Boros S, Kövér KE (2011). Low-power composite CPMG HSQMBC experiment for accurate measurement of long-range heteronuclear coupling constants: Low-power composite CPMG HSQMBC experiment. Magn Reson Chem.

[bib1.bib11] Castañar L., Parella T (2015). Broadband 
${}^{1}$
H homodecoupled NMR experiments: recent developments, methods and applications. Magn Reson Chem.

[bib1.bib12] Castañar L, Saurí J, Williamson RT, Virgili A, Parella T (2014). Pure In-Phase Heteronuclear Correlation NMR Experiments. Angew Chem Int Ed.

[bib1.bib13] Charlier C, NMRTeamTBI, Millard P (2021). Zenodo.

[bib1.bib14] Cox N, Millard P, Charlier C, Lippens G (2021). Improved NMR Detection of Phospho-Metabolites in a Complex Mixture. Anal Chem.

[bib1.bib15] Dickinson WC (1950). Dependence of the 
$F^{19}$
 Nuclear Resonance Position on Chemical Compound. Phys Rev.

[bib1.bib16] Emwas A-H, Roy R, McKay RT, Tenori L, Saccenti E, Gowda GAN, Raftery D, Alahmari F, Jaremko L, Jaremko M, Wishart DS (2019). NMR Spectroscopy for Metabolomics Research. Metabolites.

[bib1.bib17] Enthart A, Freudenberger JC, Furrer J, Kessler H, Luy B (2008). The CLIP/CLAP-HSQC: Pure absorptive spectra for the measurement of one-bond couplings. J Magn Reson.

[bib1.bib18] Foroozandeh M, Adams RW, Meharry NJ, Jeannerat D, Nilsson M, Morris GA (2014). Ultrahigh-resolution NMR spectroscopy. Angew Chem Int Ed Engl.

[bib1.bib19] Guennec AL, Giraudeau P, Caldarelli S (2014). Evaluation of Fast 2D NMR for Metabolomics. Anal Chem.

[bib1.bib20] Gutowsky HS, McCall DW (1951). Nuclear Magnetic Resonance Fine Structure in Liquids. Phys Rev.

[bib1.bib21] Helmus JJ, Jaroniec CP (2013). Nmrglue: an open source Python package for the analysis of multidimensional NMR data. J Biomol NMR.

[bib1.bib22] Jeener J, Alewaeters G (2016). “Pulse pair technique in high resolution NMR” a reprint of the historical 1971 lecture notes on two-dimensional spectroscopy. Prog Nucl Mag Res Sp.

[bib1.bib23] Karunanithy G, Mackenzie H, Hansen F (2021). Virtual Homonuclear Decoupling in Direct Detection NMR Experiments using Deep Neural Networks. ChemRxiv, Cambridge Open Engage, Cambridge, UK.

[bib1.bib24] Kessler H, Müller A, Oschkinat H (1985). Differences andsums of traces within, COSY spectra (DISCO) for the extraction of coupling constants: “Decoupling” after the measurement. Magn Reson Chem.

[bib1.bib25] Kobzar K, Skinner TE, Khaneja N, Glaser SJ, Luy B (2004). Exploring the limits of broadband excitation and inversion pulses. J Magn Reson.

[bib1.bib26] Kövér KE, Batta G, Fehér K (2006). Accurate measurement of long-range heteronuclear coupling constants from undistorted multiplets of an enhanced CPMG-HSQMBC experiment. J Magn Reson.

[bib1.bib27] Koźmiński W (1999). Simplified Multiplet Pattern HSQC-TOCSY Experiment for Accurate Determination of Long-Range Heteronuclear Coupling Constants. J Magn Reson.

[bib1.bib28] Kupče Ē (2020). Perspectives of adiabatic decoupling in liquids. J Magn Reson.

[bib1.bib29] Live D, Armitage IM, Dalgarno DC, Cowburn D (1985). Two-Dimensional 
${}^{1}$
H-113Cd Chemical-Shift Correlation Maps by 
${}^{1}$
H-Detected Multiple-Quantum NMR in Metal Complexes and Metalloproteins. J Am Chem Soc.

[bib1.bib30] Luy B, Kobzar K, Skinner TE, Khaneja N, Glaser SJ (2005). Construction of universal rotations from point-to-point transformations. J Magn Reson.

[bib1.bib31] Markley JL, Bax A, Arata Y, Hilbers CW, Kaptein R, Sykes BD, Wright PE, Wüthrich K (1998). Recommendations for the presentation of NMR structures of proteins and nucleic acids–IUPAC-IUBMB-IUPAB Inter-Union Task Group on the standardization of data bases of protein and nucleic acid structures determined by NMR spectroscopy. Eur J Biochem.

[bib1.bib32] Maudsley AA, Ernst RR (1977). Indirect detection of magnetic resonance by heteronuclear two-dimensional spectroscopy. Chem Phys Lett.

[bib1.bib33] Meissner A, Duus Jø, Sørensen OW (1997). Spin-State-Selective Excitation. Application for E.COSY-Type Measurement ofJHHCoupling Constants. J Magn Reson.

[bib1.bib34] Moreno A, Hansen KØ, Isaksson J (2019). CSSF-CLIP-HSQMBC: measurement of heteronuclear coupling constants in severely crowded spectral regions. RSC Adv.

[bib1.bib35] Morris GA, Freeman R (1979). Enhancement of nuclear magnetic resonance signals by polarization transfer. J Am Chem Soc.

[bib1.bib36] Nuzillard JM, Bernassau JM (1994). A DISCO Approach to the Measurement of Heteronuclear Long-Range Coupling Constants. J Magn Reson B.

[bib1.bib37] Oschkinat H, Freeman R (1984). Fine structure in two-dimensional NMR correlation spectroscopy. J Magn Reson.

[bib1.bib38] Pell AJ, Keeler J (2007). Two-dimensional 
J
-spectra with absorption-mode lineshapes. J Magn Reson.

[bib1.bib39] Proctor WG, Yu FC (1950). The Dependence of a Nuclear Magnetic Resonance Frequency upon Chemical Compound. Physne du Rev.

[bib1.bib40] Prud'homme SM, Hani YMI, Cox N, Lippens G, Nuzillard J-M, Geffard A (2020). The Zebra Mussel (Dreissena polymorpha) as a Model Organism for Ecotoxicological Studies: A Prior 
${}^{1}$
H NMR Spectrum Interpretation of a Whole Body Extract for Metabolism Monitoring. Metabolites.

[bib1.bib41] Purcell EM, Torrey HC, Pound RV (1946). Resonance Absorption by Nuclear Magnetic Moments in a Solid. Phys Rev.

[bib1.bib42] Ramsey NF (1950). Magnetic Shielding of Nuclei in Molecules. Phys Rev.

[bib1.bib43] Roberts MF, Vidusek DA, Bodenhausen G (1980). Adducts of ethylmercury phosphate with amino acids studied by indirect detection of 199Hg NMR. FEBS Lett.

[bib1.bib44] Saurí J, Parella T, Espinosa JF (2013). CLIP-HSQMBC: easy measurement of small proton–carbon coupling constants in organic molecules. Org Biomol Chem.

[bib1.bib45] Schätzlein MP, Becker J, Schulze-Sünninghausen D, Pineda-Lucena A, Herance JR, Luy B (2018). Rapid two-dimensional ALSOFAST-HSQC experiment for metabolomics and fluxomics studies: application to a 
${}^{13}$
C-enriched cancer cell model treated with gold nanoparticles. Anal Bioanal Chem.

[bib1.bib46] Schlippenbach T von, Oefner PJ, Gronwald W (2018). Systematic Evaluation of Non-Uniform Sampling Parameters in the Targeted Analysis of Urine Metabolites by 
${}^{1}$
H, 
${}^{1}$
H 2D NMR Spectroscopy. Sci Rep.

[bib1.bib47] Tal A, Frydman L (2010). Single-scan multidimensional magnetic resonance. Prog Nucl Mag Res Sp.

[bib1.bib48] Tiainen M, Maaheimo H, Soininen P, Laatikainen R (2010). ${}^{13}$
 C isotope effects on 
${}^{1}$
 H chemical shifts: NMR spectral analysis of 
${}^{13}$
 C-labelled D-glucose and some 
${}^{13}$
 C-labelled amino acids: 
${}^{13}$
 C isotope effects on 
${}^{1}$
 H chemical shifts. Magn Reson Chem.

[bib1.bib49] Timári I, Kaltschnee L, Raics MH, Roth F, Bell NGA, Adams RW, Nilsson M, Uhrín D, Morris GA, Thiele CM, Kövér KE (2016). Real-time broadband proton-homodecoupled CLIP/CLAP-HSQC for automated measurement of heteronuclear one-bond coupling constants. RSC Adv.

[bib1.bib50] Titman JJ, Neuhaus D, Keeler J (1989). Measurement of long-range heteronuclear coupling constants. J Magn Reson.

[bib1.bib51] Watanabe M, Meyer KA, Jackson TM, Schock TB, Johnson WE, Bearden DW (2015). Application of NMR-based metabolomics for environmental assessment in the Great Lakes using zebra mussel (Dreissena polymorpha). Metabolomics.

[bib1.bib52] Williamson RT, Márquez BL, Gerwick WH, Kövér KE (2000). One- and two-dimensional gradient-selected HSQMBC NMR experiments for the efficient analysis of long-range heteronuclear coupling constants​​​​​​​.

[bib1.bib53] Wishart DS (2019). NMR metabolomics: A look ahead. J Magn Reson.

[bib1.bib54] Wollborn U, Leibfritz D (1992). Measurements of heteronuclear long-range coupling constants from inverse homonuclear 2D NMR spectra. J Magn Reson.

[bib1.bib55] Zangger K, Sterk H (1997). Homonuclear Broadband-Decoupled NMR Spectra. J Magn Reson.

[bib1.bib56] Zhang B, Powers R, O'Day EM (2020). Evaluation of Non-Uniform Sampling 2D 
${}^{1}$
H–
${}^{13}$
C HSQC Spectra for Semi-Quantitative Metabolomics. Metabolites.

