# Supplementary material for: Virtual decoupling to break the simplification versus resolution trade-off in nuclear magnetic resonance of complex metabolic mixtures
Source: Magn Reson (Gott). 2021 Aug 10;2(2):619–27. doi: 10.5194/mr-2-619-2021 (PMC10539796; doi:10.5194/mr-2-619-2021)
Supplement: The supplement related to this article is available online at: https://doi.org/10.5194/mr-2-619-2021-supplement. [file mr-2-619-supplement.pdf]

Supplement of Magn. Reson., 2, 619–627, 2021
https://doi.org/10.5194/mr-2-619-2021-supplement
© Author(s) 2021. CC BY 4.0 License.

Supplement of

Virtual decoupling to break the simplification versus resolution trade-off in nuclear magnetic resonance of complex metabolic mixtures

Cyril Charlier et al.

Correspondence to: Cyril Charlier (charlier@insa-toulouse.fr) and Guy Lippens (glippens@insa-toulouse.fr)

The copyright of individual parts of the supplement might differ from the article licence.

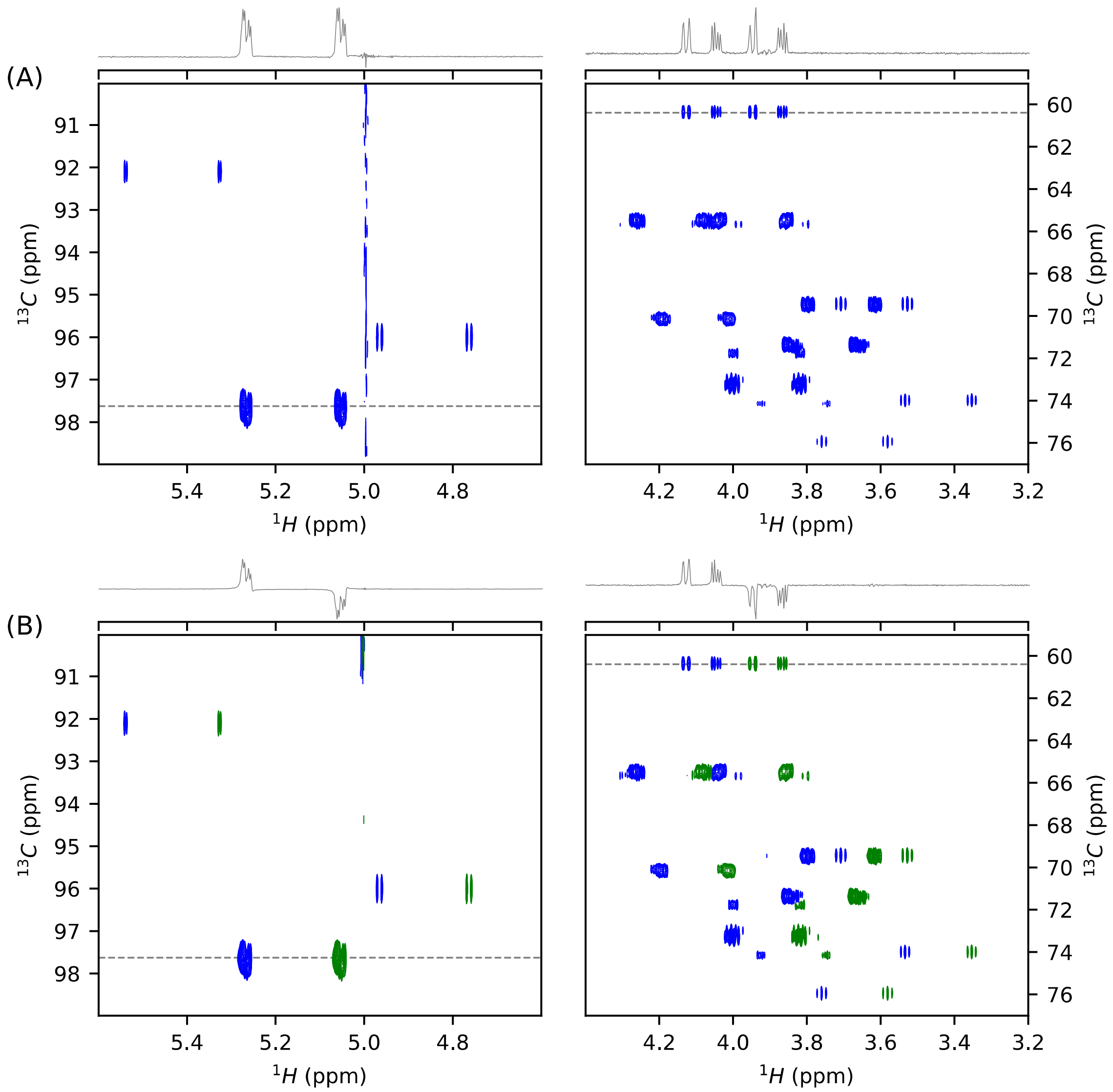

Figure S1. CLIP/CLAP HSQC spectra of the isolated dextran oligosaccharide. (A) CLIP-HSQC (B) CLAP-HSQC shown with positive contours in blue and negative in green. 1D ^{1}H spectra correspond to the traces (dotted lines) displayed on each panel.

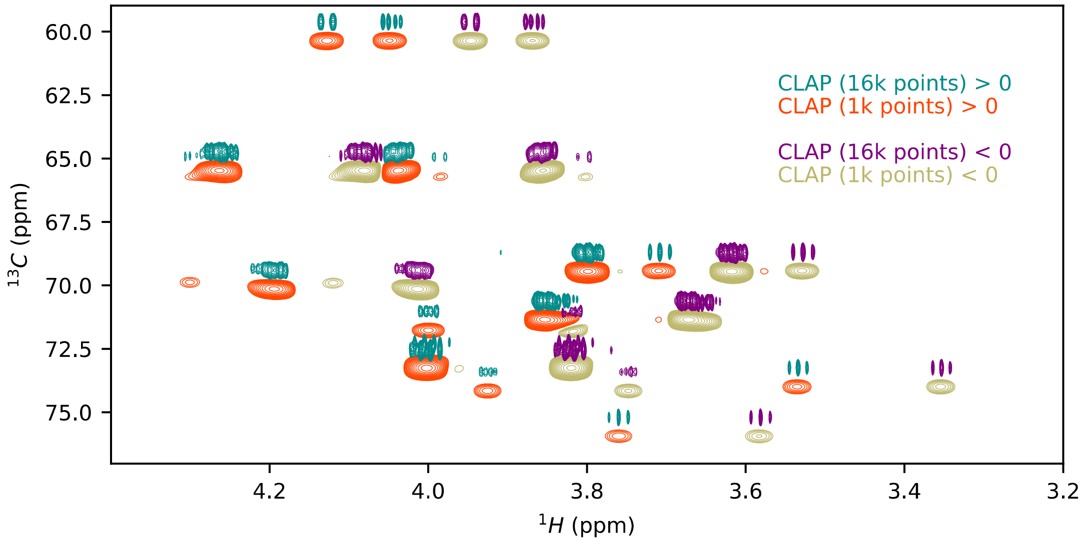

Figure S2. CLAP spectra acquired on the isolated dextran oligosaccharide processed with 16k points (cyan and purple) or with 1k points (orange and kakhi). The CLAP spectrum with 16k points was artificially shifted for visualization.

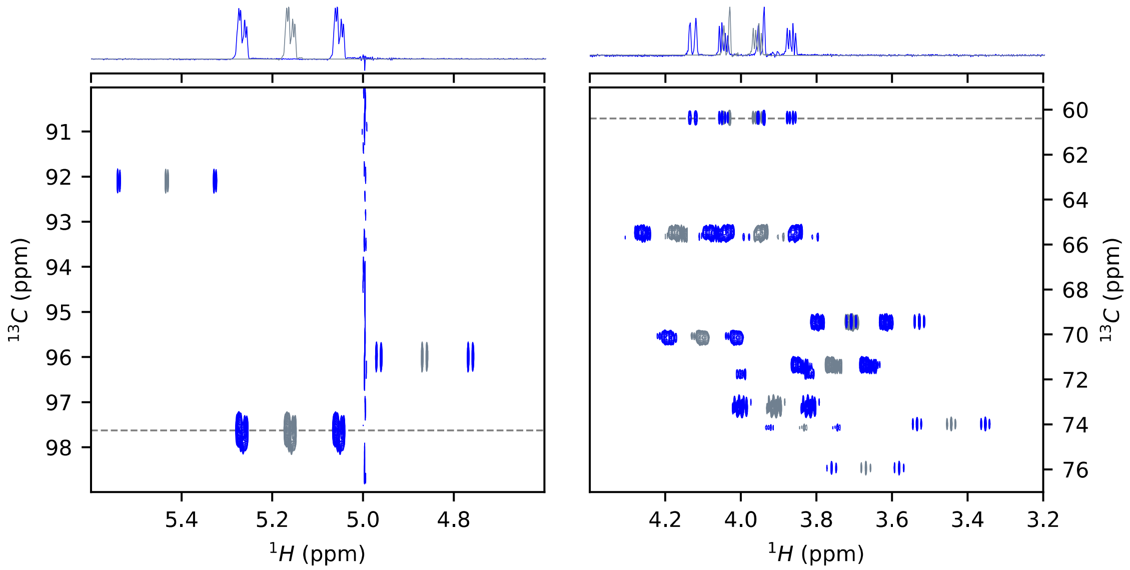

Figure S3. vd-HSQC (gray) overlay with the CLIP (blue) spectra on the isolated dextran oligosaccharide.
